# Measurement of D-dimer in cerebrospinal fluid using a luminescent oxygen channeling immunoassay

**DOI:** 10.3389/fneur.2022.951802

**Published:** 2022-10-20

**Authors:** Konstantin Kohlhase, Jan Hendrik Schaefer, Wolfgang Miesbach, Gudrun Hintereder, Konstantin Kirchmayr, Birga Zwinge, Yavor Yalachkov, Christian Foerch, Martin A. Schaller-Paule

**Affiliations:** ^1^Department of Neurology, University Hospital Frankfurt, Goethe-University, Frankfurt am Main, Germany; ^2^Department of Haemostaseology and Hemophilia Center, Medical Clinic 2, Institute of Transfusion Medicine, University Hospital Frankfurt, Goethe University, Frankfurt am Main, Germany; ^3^Central Laboratory, Centre of Internal Medicine, University Hospital Frankfurt, Goethe University, Frankfurt am Main, Germany; ^4^Punktmed Center for Vascular Medicine and Haemostaseology, Nuremberg, Germany

**Keywords:** coagulation, biomarker, diagnostics, laboratory, immunoassay

## Abstract

**Background:**

Measurement of D-dimer in cerebrospinal fluid (CSF) allows insight into coagulation system activation in the central nervous system and can be utilized to monitor intracranial hemorrhage as well as acute phase processes beyond hemostasis in inflammatory and neoplastic diseases. So far, the measurability of D-dimer in low and very low concentrations in CSF was limited in conventional immunoassays. Novel high-sensitivity chemiluminescent immunoassays such as the luminescent oxygen channeling immunoassay (LOCI^®^) are getting increasingly available but have not been validated in CSF. The aim of this study was to investigate the accuracy and linearity of the LOCI^®^ in assessing D-dimer in CSF.

**Methods:**

INNOVANCE LOCI hs D-dimer reagent cartridge was used for the measurement of D-dimer in CSF of patients with different neurological diseases. For the evaluation of linearity, dilution series were performed in a pooled CSF sample with the determination of intra-assay precision (CV, coefficient of variation) in 3 individual samples with 20 replicates. Furthermore, D-dimer concentrations measured by LOCI^®^ were compared with the respective results of a routinely available clinical latex-enhanced immunoassay (HemosiIL D-Dimer HS 500).

**Results:**

Linear regression analysis of the LOCI^®^ method revealed a *r*^2^ of 1.00 (*p* < *0*.001) with a regression coefficient B of 1.012 ± 0.003 (CI: 1.005–1.019, *p* < *0*.001) and an intercept of −1.475 ± 1.309 (CI: −4.493 to 1.543); the median intra-assay CV was 0.69% (range: 0.68–0.75). In total, 185 CSF samples were measured by LOCI^®^ technology, showing a mean concentration of 204.84 ± 2,214.93 ng/ml. D-dimer concentration between LOCI and latex-enhanced immunoassay differed by a factor of 10.6 ± 13.6 on average with a maximum deviation by a factor of 61.3; the maximum deviation was found at low concentrations.

**Conclusion:**

D-dimer in CSF of patients with neurological disease can be reliably measured by the LOCI^®^ method with high linearity and accuracy at low concentrations.

## Introduction

Measurement of D-dimer in cerebrospinal fluid (CSF) allows insights into coagulation cascade activation in the central nervous system to monitor different aspects of intracranial hemorrhage, as well as other acute phase processes beyond hemostasis in inflammatory and neoplastic diseases (1, 2). Due to a disturbance of the blood-brain barrier, clotting factors such as fibrinogen enter the cerebrospinal fluid (CSF), where it is converted into cross-linked fibrin and degraded into breakdown products such as D-dimer in the further course (3, 4). Besides D-dimer, other components of the coagulation cascade such as procoagulant phospholipids (PPLs), prothrombin fragments, and tissue factors have already been successfully detected in CSF (5, 6). While the measurement of D-dimer in citrated blood to rule out acute deep-vein thrombosis with latex-immunoassays represents an established, well-validated laboratory analysis, the measurement of D-dimer in CSF is so far limited to scientific work (7, 8). Among the available studies, D-dimer measurements in CSF were mostly performed by (automated) enzyme-linked immunosorbent assays (ELISA) or latex-based immunoassays (6, 9–12). However, the field of application of the commonly used automated assays, which have been validated in blood for the exclusion of acute thrombosis, e.g., latex-enhanced immunoassays, revealed a decreasing accuracy and linearity for low concentrations <200 ng/ml (8, 13). While (conventional) ELISAs are feasible to also detect lower concentrations, these carry disadvantages for the clinical routine of being more time-consuming, expensive, and potentially susceptible to interferences (14, 15). However, low D-dimer concentrations in CSF represent an area of interest for diseases such as multiple sclerosis, where an increase in D-dimer revealed a positive correlation with markers of acute inflammation (16). Chemiluminescence-based immunoassays such as the homogeneous, automated luminescent oxygen channeling immunoassay (LOCI^®^) are reported to exhibit a high diagnostic range and high accuracy in the measurement of D-dimer in blood with a potentially lower limit of detection than common immunoassays, thus representing an advantageous alternative for the measurement of D-dimer in CSF (17). To the best of our knowledge, a validation of the LOCI^®^ method for the measurement of D-dimer in CSF has not yet been performed.

In this study, we present our experience with the measurement accuracy of the LOCI^®^ method for the detection of D-dimer in CSF in a cohort of patients with primary CNS disease. Furthermore, we compare these results with a conventional and widely used latex-based immunoassay (HemosIL D-Dimer HS 500), which represented the reference standard for detecting D-dimer in blood plasma at our hospital.

## Methods

### Patient acquisition

This study was conducted at the Department of Neurology, Goethe University, Frankfurt am Main, and was approved by the Institutional Review Board of the University Hospital Frankfurt (project number: 173/19). Samples were obtained from the Biobank of Chronic Inflammatory CNS Diseases, which were collected between October 2017 and December 2020. The samples were derived from patients older than 18 years of age who underwent a clinically indicated lumbar puncture due to suspected primary CNS disease. Final diagnoses according to the ICD-10 classification [International Statistical Classification of Diseases and Related Health Problems (ICD), 10th revision] (18) included patients with multiple sclerosis [analogous to the 2017 McDonald criteria revision (19)], myelin oligodendrocyte glycoprotein antibody-associated disease (MOGAD), neuromyelitis optica spectrum disorders (NMOSD) with antibodies to aquaporin-4, other acute CNS inflammation (e.g., bacterial/viral meningitis), CNS neoplasia, non-CNS neurologic disease, other diseases, and no evidence of neurological disease. Written informed consent was mandatory before inclusion and was provided by the patients or their legal representative.

### CSF acquisition

During diagnostic lumbar puncture, 2.6 ml of CSF was collected in a citrate plasma tube (S-Monovette 2.6 ml LH-GEL+, Sarstedt AG & Co. KG) to avoid coagulation of the clotting factors. Specimens with visible blood staining due to traumatic puncture were excluded. The citrated CSF was centrifuged at 3,000 rpm for 10 min, pipetted, and frozen at −80°C. The measurement of D-dimer was performed using 500 μl citrated CSF. For the measurement of D-dimer, the samples were thawed to a temperature of 37°C using a ThermoMixer C (Eppendorf AG, Hamburg, Germany).

### Luminescent oxygen channeling assay (LOCI)

Quantitative measurement of D-dimer was performed in citrated CSF using the INNOVANCE LOCI hs D-dimer reagent cartridge (Siemens Healthineers, Erlangen, Germany), which is an automated immunoassay based on the luminescent oxygen channeling immunoassay (LOCI^®^) technology. The analysis was performed on the fully automated Atellica COAG 360 System (Siemens Healthineers, Erlangen, Germany). The LOCI^®^ technology represents a homogeneous bead-based sandwich chemiluminescent immunoassay. The assay contains two synthetic latex beads (sensibead and chemibead) and a biotinylated anti-D-dimer antibody. The sensibead is coated with streptavidin and a photosensitizer dye, whereas the chemibead is coated with anti-D-dimer antibodies and an additional chemiluminescent dye. In the first step, the sample with D-dimer is added to the chemibead and the biotinylated antibodies, resulting in the formation of an immune complex across the antibody binding sites. This step is followed by the addition of the sensibead, resulting in the formation of the bead-pair immune complex. Subsequent illumination of the bead-pair immune complex at a wavelength of 680 nm forms singlet oxygen within the sensibead that diffuses to the adjacent chemibeads to trigger a chemiluminescent signal that is measured at 612 nm and is proportional to the concentration of D-dimer in the sample (20). The linearity assessment was performed in a pooled human CSF sample of 20 different patients. A pooled CSF was used to avoid interferences from different CSF compositions of individual patients. To cover a wide range of D-dimer concentrations for linearity analysis, the pooled sample was mixed with the manufacturer's D-dimer quality control (INNOVANCE LOCI^®^ Control 2, Siemens Healthineers, Erlangen, Germany) at a targeted D-dimer concentration of 1,000 ng/ml. Dilution series were performed using INNOVANCE D-dimer diluent (Siemens Healthineers, Erlangen, Germany). Intra-assay precision, given as coefficient of variation (CV), was calculated in 3 samples with 20 replicates each, as well as for the duplicate measurements of the linearity assessment.

### Latex-enhanced immunoassay

HemosIL D-Dimer HS 500 assays (Werfen, Munich, Germany) are fully automated, latex-enhanced turbidimetric immunoassays consisting of a latex reagent in a suspension of polystyrene latex particles with a highly specific binding domain of F(ab')2 antibody fragments (MA-8D3) for D-dimer. The administration of D-dimer in the analyte causes agglutination of the latex particles, which is proportional to the concentration of D-dimer. Agglutination results in turbidity of the suspension, which can be quantified by the transmission of light with a wavelength of 671 nm. The measurement was performed on the ACL TOP 700 analyzer (Werfen, Munich, Germany). Dilution series for linearity assessment were performed using the factor diluent (Werfen, Munich, Germany).

### Statistics

Statistical analysis was performed using IBM SPSS^®^ (IBM Corp. Released 2021. IBM SPSS Statistics for Macintosh, Version 28.0. Armonk, NY: IBM Corp). The accuracy of linearity in dilution was determined using linear regression analysis to calculate r^2^ and the respective regression equation. Intra-assay precision expressed as the coefficient of variation (CV) between duplicate measurements was calculated by the following equation: standard deviation of duplicate^*^100/duplicate mean. Since intra-assay precision did not reveal normal distribution, CV was given as the median with the first and third quartiles (Q1-Q3). Wilcoxon test for dependent variables was used to assess differences between D-dimer concentrations measured by LOCI^®^ technology and conventional latex immunoassays. A *p*-value < 0.05 was determined to be significant.

## Results

### Linearity analysis of D-dimer in CSF using LOCI^®^

**Figure 1:**
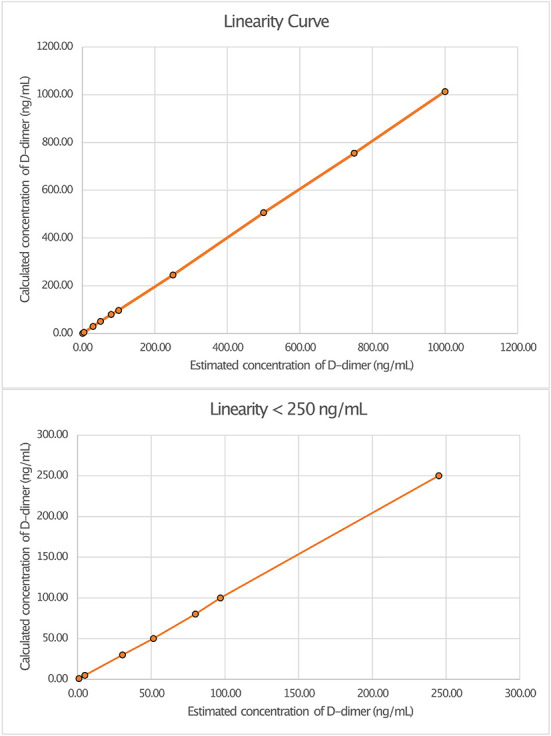
Linearity curves of D-dimer in cerebrospinal fluid (CSF) measured with luminescent oxygen channeling immunoassay (LOCI^®^) INNOVANCE LOCI hs D-dimer assay.

**Figure 2:**
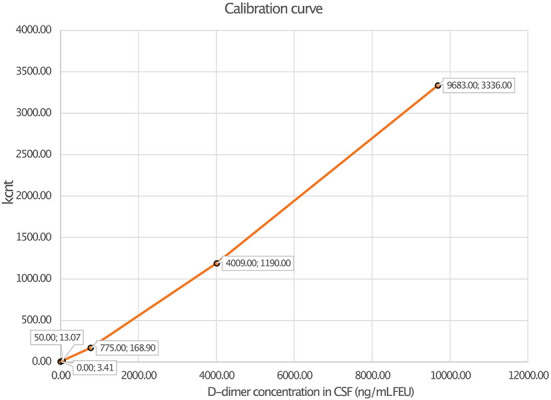
Calibration curve of D-dimer measurement in cerebrospinal fluid (CSF) using luminescent oxygen channeling immunoassay (LOCI^®^) INNOVANCE LOCI hs D-dimer assay.

Linearity analysis of the INNOVANCE LOCI hs D-dimer assay was performed in a pooled human CSF sample with the addition of the manufacturer's D-dimer reagent at a target concentration of 1,000 ng/ml; the pooled sample consisted of 68.2% CSF and 31.8% D-dimer reagent. For the assessment of linearity, dilution series were prepared at dilutions of 0.1, 0.5, 3, 5, 8, 10, 25, 50, 75, and 100% with subsequent measurements as duplicates (Figure 1, Table 1). Linear regression analysis revealed a r^2^ of 1.00 (*p* < *0*.001) with a regression coefficient B of 1.012 ± 0.003 (CI: 1.005–1.019, *p* < *0*.001) and an intercept of −1.475 ± 1.309 (CI: −4.493 to 1.543), corresponding to a regression equation of y = −1.47 + 1.01^*^x. Intra-assay CV between duplicates of the linearity measurements showed a median of 0.3% (Q1–Q3: 0.0–1.05%) with a maximum CV of 2.3%. The calibration curve was prepared at dilutions of 0:1, 1:231, 1:15, 1:2.9, and 1:1.2 (Figure 2). Intra-assay precision of the 3 samples with 20 replicates was in a median of 0.69% (range: 0.68–0.75).

**Table 1:** Measurements of D-dimer in CSF by luminescent oxygen channeling immunoassay (LOCI^®^) in different dilution series.

**Dilution**	**Measurement 1 (ng/ml)**	**Measurement 2 (ng/ml)**	**Mean concentration (ng/ml)**	**CV (%)**	**Estimated concentration (ng/ml)**
0.1 %	1.0	1.0	1.0	0.00	1.0
0.5 %	5.0	5.0	5.0	0.00	5.0
3 %	31.0	30.0	30.5	2.32	30.0
5 %	51.0	52.0	51.5	1.37	50.0
8 %	80.0	80.0	80.0	0.00	80.0
10 %	97.0	97.0	97.0	0.00	100.0
25 %	243.0	247.0	245.0	1.15	250.0
50 %	505.0	508.0	506.5	0.42	500.0
75 %	754.0	756.0	755.0	0.19	750.0
100 %	1,019.0	1,008.0	1,013.5	0.77	1,000.0

The measurements were performed as duplicates (Measurements 1 and 2). CV, coefficient of variation.

### Linearity analysis of D-dimer in CSF using HemosIL HS 500 immunoassay

**Figure 3:**
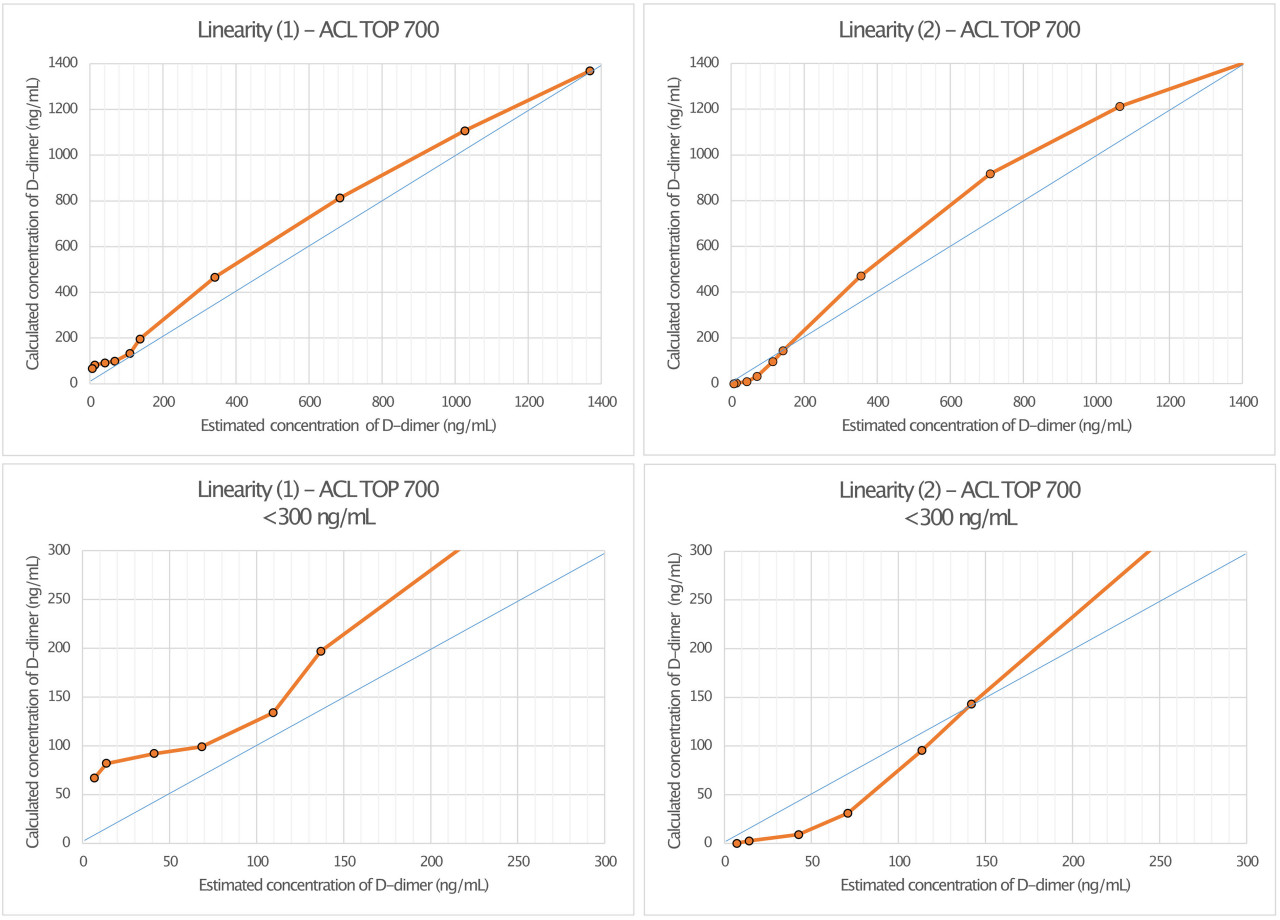
Linearity assessment of D-dimer measurement in cerebrospinal fluid (CSF) using HemosIL HS 500 reagent latex-enhanced immunoassay. The measurement was performed on two different ACL TOP 700 analyzers [Linearity (1) and Linearity (2)].

Linearity assessment of the HemosIL HS 500 assay was performed on two different ACL TOP 700 analyzers (“1” and “2”) using a sample of a male patient with relapsing-remitting multiple sclerosis. Dilution series were performed at the concentrations 0.5, 1, 3, 5, 8, 10, 25, 50, 75, and 100%. Linear regression analysis on ACL TOP Analyzer 1 showed a r^2^ of 0.993 (*p* < *0*.001) with a regression coefficient B of 0.996 ± 0.03 (CI: 0.927–1.065, *p* < *0*.001) and an intercept of 64.44 ± 17.81 (CI: 22.38–105.50), corresponding to a regression equation of y = 64.44 + 0.996^*^x. For ACL TOP Analyzer 2, r^2^ was 0.98 (*p* < *0*.001) with a regression coefficient B of 1.083 ± 0.054 (CI: 0.957–1.208, *p* < *0*.001), an intercept of 2.68 ± 33.6 (CI: −75.795 to 80.148), and a regression equation of y = 2.68 + 1.08^*^x (Figure 3).

### Comparison of D-dimer concentration between INNOVANCE LOCI^®^ and HemosIL HS 500 immunoassay

**Figure 4:**
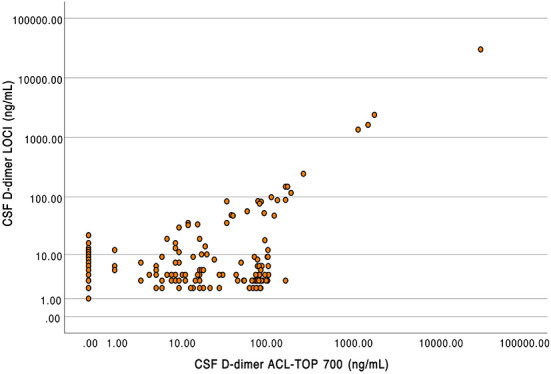
Scatterplot of D-dimer concentrations in cerebrospinal fluid measured using luminescent oxygen channeling immunoassay (LOCI, y-axis) and latex-enhanced turbidimetric immunoassays (HemosIL HS 500 reagent) on the ACL TOP 700 (x-axis).

A total of 185 patient samples were available in which D-dimer in the CSF was measured by both INNOVANCE LOCI^®^ hs D-dimer and the HemosIL HS 500 assay. D-dimer averaged 204.84 ± 2214.93 ng/ml when measured by LOCI^®^ and 238.29 ± 2395.66 ng/ml when measured by the latex-enhanced immunoassay. There were significant differences between the measured values between LOCI^®^ and latex-enhanced immunoassay (Wilcoxon test: Z = −5.031, *p* < 0.001). D-dimer concentration measured by LOCI and latex-enhanced immunoassay revealed a deviation by a factor of 10.6 ± 13.6 on average with a maximum deviation by a factor of 61.3; values with a concentration of 0 ng/ml were excluded from this particular calculation (*n* = 62). A scatterplot depicting the respective concentrations between the different measurements is shown in Figure 4.

## Discussion

The findings of this study demonstrate that the LOCI^®^ method and INNOVANCE LOCI hs D-dimer assay enable a reliable measurement of D-dimer in CSF.

Linearity in dilution was obtained over the entire measured range with the LOCI^®^ method, whereby concentrations as low as 1 ng/ml could reliably be detected (Figure 1). In general the LOCI^®^ method is reported to show high robustness and sensitivity with low susceptibility to interference effects in blood plasma (21). One reason for the exact measurement might be the usually lower required concentrations of receptor-coated particles in the LOCI method compared to conventional latex immunoassays as well as the chemiluminescence technology, in which the illumination induced singlet oxygen diffuses into solution but typically only activates its own particle-complex due to its short half-life so that the risk of activation of unbound particles with false high-values is negligible (21). In accordance with this, a study by Kappel and Ehm demonstrated high sensitivity for the measurement of D-dimer in blood by LOCI^®^ with an assay range of 26–3.094 ng/ml and high linearity in dilution in the regression analysis (r = 0.999) with a within-run precision of <1.2% and a total precision of 3.2% (17). Similar results to the values in blood were demonstrated in CSF, with linearity in the regression analysis of r^2^ = 1.0 and a CV in a median of 0.3% and a maximum of 2.3%. Although interference of the CSF with the binding to the antibodies may occur due to the CSF itself or due to components such as albumin, interfering antibodies, or blood components, this did not seem to be a relevant problem for the measurement by the LOCI^®^ method in our study (22).

The expected concentration of D-dimer in CSF depends on the underlying disease and mechanism of fibrin degradation. Naturally, very high concentrations of D-dimer in CSF are reported by Chen et al. (10) and Van Dreden et al. (6) in patients with intracranial hemorrhage (820 ng/ml [520–980 ng/ml] to 43,100 ± 45,800 ng/ml) communicating with the subarachnoid space or the ventricular system (43,100 ± 45,800 ng/ml) (6, 10). In these cases, D-dimer derives directly from blood clots inside the respective CSF compartment. Besides intracranial hemorrhages, patients with bacterial meningitis {2,200 ng/ml [400–4,700 ng/ml], Weisfelt et al. (11)} or meningeosis carcinomatosa are reported to show a massive increase in D-dimer, which is suspected to result from a disturbance of the blood-brain barrier with fibrinogen transfer into CSF, which is subsequently converted by thrombin into cross-linked fibrin with an increase in D-dimer in the further course of fibrin degradation (3, 11, 16). The accumulation of degradation products such as D-dimer appears to increase with the extent of blood-brain barrier disruption, which can be severe in neoplastic or infectious diseases (16, 23, 24). However, also pyogenic vasculitis with microbleeds may be another contributing factor for increased D-dimer in CSF during bacterial meningitis (25). In addition, in meningeosis carcinomatosa pro-coagulant factors may be released, for example, through the tumor cells themselves, which facilitates the formation of D-dimer (26). Furthermore, chronic inflammatory CNS diseases such as relapsing-remitting multiple sclerosis (median 4 ng/ml [IQR 3–8]) were shown to cause a minor to moderate increase of D-dimer in CSF compared to controls and during signs of acute inflammation on MRI (median 6 ng/ml [IQR 3–15.25]) (16).

Compared to the LOCI^®^ method, latex immunoassays such as the HemosIL HS 500 on the ACL TOP 700 showed a significant decrease in linearity and measurement accuracy at concentrations of 150–200 ng/ml, matching the reference values available from blood (27). In addition, differences also appeared between the two ACL TOP 700 analyzers used for the measurements, such that false-high values were measured in analyzer (I) and false-low values were measured in analyzer (II). Since the same CSF and factor diluent were used for the measurements, we considered this to be due to different calibrations of the devices (28). D-dimer concentrations measured using the LOCI^®^ technique showed a discrepancy by the factor of up to 61.3 compared to the results of the HemosIL HS 500. In the scatter plot (Figure 4), this is visualized by a broad spread of the measured values, especially in the range of concentrations below 200 ng/ml, which could be either too low or too high. As the concentration of D-dimer increases, this spread decreases and becomes more linear. Interferences with blood components such as hemoglobin, bilirubin, triglycerides, or rheumatoid factor have been described for the HemosIL HS 500 (8). Since these components do not occur in relevant concentrations in CSF, and CSF with visible blood staining was excluded, we suggest technical limitations as the reason for the decreasing accuracy at low concentrations instead of relevant interferences (29).

## Limitations

Among the available methods, quantitative ELISAs so far represented the gold standard for the determination of plasma D-dimer. While conventional ELISAs are reported to show a high sensitivity to detect D-dimer, they bear the disadvantage of time-consuming processing, which is why they are rarely used routinely in clinical laboratories. Among automated ELISAs, highly sensitive assays such as the VIDAS^®^ D-Dimer Exclusion II™ (bioMérieux, Marcy-l'Étoile, France) are available. However, since automated ELISAs have often been developed for routine exclusion of deep vein thrombosis or pulmonary artery embolism, they commonly show a decreasing sensitivity in low D-dimer concentrations (e.g., <45 ng/ml) (30). In our study, we aimed to focus on the measurement accuracy of the LOCI method and present our experience in measuring D-dimer in CSF. Since conventional ELISAs were not routinely available and do not allow a representative comparison with techniques available in common clinical practice, we compared LOCI^®^ measurement to our in-house reference device for routine determination of D-dimer, a latex-immunoassay. Nevertheless, this also represents a limitation of our study since no head-to-head comparison with the current gold standard for the determination of D-dimer was made, which would be desirable for the evaluation of the significance of the LOCI method in the future. A further limitation of the study was the different compositions of the samples for the respective linearity measurement. While a pooled sample was used for the LOCI^®^ method, this was done on the ACL TOP 700 using a single selected sample. One advantage of a pooled sample is the reduction of individual components of the CSF between different patients, which could probably interfere with the assay. Since the decreasing linearity of the ACL TOP 700 corresponds to the data in blood, we consider these to be representative results. However, since two different samples were used for the measurement performed by latex-enhanced immunoassay and LOCI^®^ method, differences regarding the samples in terms of possible interferences cannot be entirely excluded. Furthermore, duplicate measurements of patient samples using the LOCI^®^ method were not available in every measurement, so an assessment of the precision over all measured samples was not possible.

In summary, the LOCI^®^ method and latex-enhanced turbidimetric immunoassays were able to quantify D-dimer in CSF, with the LOCI^®^ method exhibiting advantages in measurement accuracy over immunoassays such as the HemosIL HS 500, particularly at low concentrations. Considering the expected concentration ranges of D-dimer in CSF depending on the disease spectrum, the adequate method for measurement can be weighed against each other. This allows the LOCI method to provide additional benefit in diseases such as chronic inflammatory CNS disease or intracranial hemorrhage with only minor blood passage into the subarachnoid space.

## Conclusion

The findings of this study indicate that D-dimer in CSF can be detected using the LOCI^®^ method with a high linearity and measurement accuracy even at low concentrations.

## Data availability statement

Anonymized data not published within this article will be made available by request from any qualified investigator.

## Ethics statement

The studies involving human participants were reviewed and approved by Institutional Review Board of the University Hospital Frankfurt (project-number: 173/19). The patients/participants provided their written informed consent to participate in this study.

## Author contributions

KKo and MS-P conducted the study and performed the conceptualization, methodology, and formal analysis. KKi contributed to the methodology, formal analysis, and generation of data. JS, YY, and MS-P contributed to the data collection. WM, KKi, GH, and BZ contributed to providing the resources (measuring devices). CF supervised the study. KKo and MS-P wrote the original draft of the manuscript. JS, WM, GH, KKi, BZ, YY, and CF reviewed and edited the manuscript. All authors contributed to the manuscript and accepted the final version of the manuscript.

## Funding

This research project was supported by Sanofi Genzyme within the following study: Identification of a CSF- and blood biomarker finger-print differentiating between highly active and moderate/mild forms of multiple sclerosis (GZ-2016-11612). Siemens Healthineers granted discounts on INNOVANCE LOCI test kits for use in this study. The funders were not involved in the study design, collection, analysis, interpretation of data, the writing of this article, or the decision to submit it for publication.

## Conflict of interest

The authors declare that the research was conducted in the absence of any commercial or financial relationships that could be construed as a potential conflict of interest.

## Publisher's note

All claims expressed in this article are solely those of the authors and do not necessarily represent those of their affiliated organizations, or those of the publisher, the editors and the reviewers. Any product that may be evaluated in this article, or claim that may be made by its manufacturer, is not guaranteed or endorsed by the publisher.
